# Deciphering the role of immunoglobulin secreting malignant lineages in the invasive frontiers of small cell lung cancer by scRNA-seq and spatial transcriptomics analysis

**DOI:** 10.1038/s41421-023-00621-4

**Published:** 2023-12-12

**Authors:** Fei Wu, Xiao Zhang, Minglei Wang, Jingxin Zhang, Minxin Chen, Ziyuan Ren, Meng Wu, Pingping Song, Jinming Yu, Dawei Chen

**Affiliations:** 1 Department of Radiation Oncology and Shandong Provincial Key Laboratory of Radiation Oncology, Shandong First Medical University and Shandong Academy of Medical Sciences, Jinan, Shandong China; 2 Department of Urology, Shandong Provincial Hospital Affiliated to Shandong First Medical University, Jinan, Shandong China; 3 Department of Thoracic Surgery, Shandong Cancer Hospital and Institute, Shandong First Medical University and Shandong Academy of Medical Sciences, Jinan, Shandong China; 4 Research Unit of Radiation Oncology, Chinese Academy of Medical Sciences, Jinan, Shandong China

**Keywords:** Cancer microenvironment, Small-cell lung cancer

Dear Editor,

Lung cancer remains the leading cause of cancer-related death worldwide^1^. Small cell lung cancer (SCLC) comprises ~14% of lung cancer and causes 200,000 deaths globally^2^. SCLC is characterized by the development of treatment resistance and the early-onset of widespread metastasis. Despite numerous clinical trials conducted over the past decades, the prognosis for SCLC patients has not significantly improved, except for the recent success of IMpower133 and CASPIAN trials employing immunotherapy strategies^3,4^. However, the specific mechanism of metastasis in SCLC and its tumor immune microenvironment remain largely unknown. The current classification of SCLC into molecular subsets based on the expression of four transcription factors (ASCL1, NEUROD1, POU2F3, and YAP1) has not effectively predicted prognosis or treatment response^5^. The ASCL1-high SCLC is the most common subtype of SCLC^5^. Hence, we hypothesize that the current subtyping of SCLC has significant intratumor heterogeneity. Single-cell RNA sequencing (scRNA-seq) is a powerful tool for cancer research to understand intratumor heterogeneity and tumor immune microenvironment^6^. However, the lack of spatial information limits the interpretation of cellular heterogeneity and cell–cell interactions. Here, spatial-resolved transcriptomics and multi-regional scRNA-seq were used to investigate the diversity of cancer cells in ASCL1-high SCLC collectively.

Emerging evidence suggested that the production of immunoglobulin (Ig) has been detected in various cancer cells, such as lung adenocarcinoma and macrophages^7,8^. Cancer-derived Ig has been implicated in promoting tumor proliferation and facilitating immune escape^8^. In the context of non-SCLC, the presence of tumor-derived Ig has been associated with poor prognosis^9^. However, the presence and mechanistic significance of Ig-expressing malignant cells in SCLC have yet to be elucidated.

In this study, tissues at five distinct locations (Core: tumor core; Intermediate: intermediate position; Margin: tumor invasive margin; Adjacent: adjacent tissues; Normal: distant normal lung tissues) were collected from three SCLC patients who underwent lung lobectomy (Fig. 1a). These tissue samples were pathologically confirmed to belong to the ASCL1-high subtype. A total of 18 samples were qualified for scRNA-seq (*n* = 10) with the 10х Genomics Chromium System and spatial-resolved transcriptomic sequencing (*n* = 8) via 10× Genomics Visium platform (each section has 5000 spots in the 6.5 mm by 6.5 mm capture area) (Fig. 1a). Single-cell transcriptomes from a total of 109,462 cells from Core (*n* = 15,864), Adjacent (*n* = 16,512), Intermediate (*n* = 31,587), Margin (*n* = 19,391), and Normal (*n* = 26,108) were obtained after initial quality controls (Fig. 1b; Supplementary Figs. S1, S2a). After annotating major cell types using canonical biomarkers, the cells derived from epithelial cells were analyzed with the InferCNV algorithm to identify malignant cells based on copy number variations. As a result, 37,535 cells were identified as malignant cells (Fig. 1b, c; Supplementary Fig. S2b–d). The multi-regional scRNA-seq of SCLC tumors suggested the remarkable heterogeneity in both tumor and benign regions (Supplementary Fig. S2e, f). Interestingly, we found that the genes encoding Ig were highly expressed in malignant cells and B cells (Fig. 1c; Supplementary Fig. S1d). To gain a comprehensive understanding, we examined the expression of multiple Ig-coding genes and the proliferation marker TOP2A across all cell types (Fig. 1d). Interestingly, besides B cells, we found that *IGKC, IGLC2, IGHG3*, and *IGHA1* were highly expressed in malignant cells and a subset of myeloid cells, while showing negligible expression in fibroblast or endothelial cells (Fig. 1d). This observation highlights the significant expression of Ig-coding genes specifically in malignant cells.

**Fig. 1:**
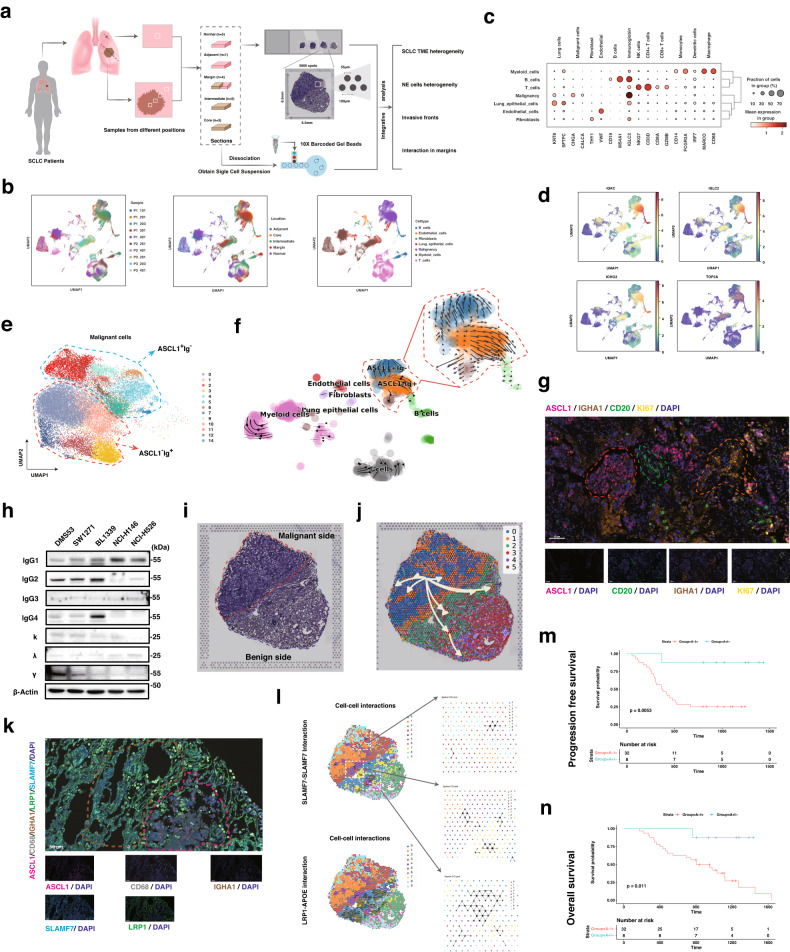
The identification and role of the immunoglobin-secreting malignant cells in the invasive frontiers of SCLC. **a** Schematic workflow and grouping information for scRNA-seq using the 10× Chromium platform and spatial transcriptomics analysis. **b** Uniform Manifold Approximation and Projection (UMAP) plot of Louvain clustering (Scanpy) of jointly analyzed single-cell transcriptomes from 109,462 cells from SCLC specimens. **c** Distribution of canonical molecular markers for indicated subpopulations visualized in the dot plot. **d** Expression of selected Ig genes (IGKC, IGLC2, and IGHG3) and proliferation markers (TOP2A) among various cells of SCLC visualized in feature plot. **e** UMAP embedding of jointly analyzed scRNA-seq from malignant cells annotated by cell type. **f** Velocities derived from the dynamical model for various cells in SCLC specimens are visualized as streamlines in a UMAP-based embedding. **g** Representative image of multiplexed immunofluorescence staining (mIF) for ASCL1 (red), CD20 (orange), IGHA1 (brown), KI67 (yellow), and DAPI (blue) in SCLC patients (×200 magnification). **h** The expression pattern of IgG1, IgG2, IgG3, IgG4, free light chains κ, λ, and heavy chains γ in SCLC cell lines (DMS53, SW1271, NCI-H1339, NCI-H146, and NCI-H526). **i** Hemoxylin and eosin (H&E) staining and spatial feature plot (IGKC, IGLC2, and IGHG3) of the sections with invasive frontiers from SCLC patients. **j** Spatial trajectory inference analysis of cluster 1 spots using stlearn algorithm showing the intratumor heterogeneity of SCLC tumor cells with spatial information. **k** Multiplexed IF staining (ASCL1, CD68, IGHA1, LRP1, SLAMF7, and DAPI) showing co-aggregation of LRP1-high macrophages (LRP1^+^CD68^+^ cells) and SLAMF7-high tumor cells (SLAMF7^+^IGHA1^+^ cells) at invasive fronts of SCLC patients. **l** Spatial cell–cell interaction analysis of invasive frontiers of an SCLC patient using stlearn. Representative receptor and ligand pairs are visualized and zoomed. Progression-free survival (**m**) and overall survival (**n**) curves of 40 patients with SCLC from our validation cohort with bulk-RNA sequencing information, which were grouped by the expression of ASCL1 and IGHA1.

Subsequently, we conducted an unsupervised clustering analysis of the malignant cells and identified distinct cell types based on top-ranked marker genes (Fig. 1e). Although the patients were initially classified to ASCL1-high subtypes of SCLC based on the overall expression of biomarkers (ASCL1, NEUROD1, POU2F3, and YAP1), a considerable proportion of cells failed to express ASCL1 (Supplementary Fig. S2g). Interestingly, the ASCL1-low malignant cells showed elevated levels of Ig genes including *IGHG3* and *IGKC* (Supplementary Fig. S2g). Data from this scRNA-seq analysis indicated that the expression of ASCL1 and Ig genes were prone to be mutually exclusive. To further verify this finding, we found that the ASCL1-high subpopulations (ASCL1^+^Ig^–^) and Ig-high cells (ASCL1^–^Ig^+^) had different transcription factors as well as the profile of target genes (Supplementary Fig. S3a–c). By comparing un-spliced and spliced mRNAs, we observed that both ASCL1^+^Ig^–^ and ASCL1^–^Ig^+^ cells were likely derived from common precursor cells but exhibited opposite developmental trajectories (Fig. 1f). Furthermore, the signaling pathways enriched in ASCL1^+^Ig^–^ and ASCL1^–^Ig^+^ were remarkably different according to the analysis of differentially expressed genes, hdWGCNA, RNA velocity, and cell–cell communications (Supplementary Figs. S3d–j, S4a–c). Notably, the neuron differentiation pathway was significantly enriched in the “turquoise” module, which exclusively correlated with the ASCL1^+^Ig^–^, but not ASCL1^–^Ig^+^ cells (Supplementary Fig. S3f–i). To further investigate the potential mutual exclusivity of ASCL1 and Ig expression in clinical specimens, we performed immunohistochemistry (IHC) and multiplexed immunofluorescence (mIF) analyses on sections from ASCL1-high SCLC patients. The regions with ASCL1 high expression were not overlapped with the Ig-high regions in IHC and mIF staining analysis (Fig. 1g; Supplementary Fig. S4d). Furthermore, the expression of IGHA1 coincided with proliferation biomarker KI67 but not CD20/MS4A1, the canonical marker gene for mature B cells (Fig. 1g). The secretion of Ig from SCLC cells was confirmed by the immunoblot of cell lines derived from SCLC (Fig. 1h). To this end, the newly identified ASCL1^–^Ig^+^ cells had distinct transcriptomic and spatial characteristics with ASCL1^+^Ig^–^ in SCLC.

Since our multi-regional scRNA-seq analysis showed the spatial heterogeneity of malignant cells, we next sought to investigate the distribution of ASCL1^–^Ig^+^ cells and their specific role in the metastasis of SCLC. Based on spatial transcriptomics (ST) analysis, ASCL1-high spots were mainly in the tumor core and intermediate regions, while the Ig-high spots were mainly accumulated at the malignant sides of tumor margins (Supplementary Fig. S5). With unsupervised clustering of spots based on transcriptomics information, the distribution of ASCL1-high spots (corresponding to the ASCL1^+^Ig^–^ cells in scRNA-seq data) and Ig-high spots (corresponding to the ASCL1^–^Ig^+^ cells in scRNA-seq data) in tumor margins were highly organized alongside the borderline between malignant and benign sides (Supplementary Fig. S5b). Since the interface at the tumor boundary could possess a conserved mechanism of tumor–microenvironment interactions in human cancer, the ST samples with invasive frontiers were selected for further analysis (Supplementary Fig. S6)^10^. To this end, the spots of tumor margin sections were analyzed by a combinatorial approach (stlearn, v0.4.8), integrating spatial distance, tissue morphology, and gene expression information from ST data (Supplementary Fig. S6a). Accordingly, all the spots were clustered into six subpopulations, where cluster 1 was annotated as ASCL1^–^Ig^+^ dominant spots based on its location at the tumor side and the distribution of Ig-high regions (Fig. 1i–j; Supplementary Fig. S6b). Notably, the ASCL1^–^Ig^+^ dominant spots (cluster 1), but not the ASCL1^+^Ig^–^ dominant spots (cluster 0), were distributed alongside the invasive fronts of SCLC (Fig. 1j; Supplementary Fig. S6b). The primary cell types in the benign sides of the interface were macrophage (cluster 2) and alveolar epithelial cells (cluster 3) (Fig. 1j; Supplementary Fig. S6a, b). Spatial trajectory inference indicated that ASCL1^–^Ig^+^ prevalent spots were highly heterogeneous, derived from the inner tumor, but could develop and migrate to regions at the benign side of invasive fronts and adjacent normal tissues (Fig. 1j; Supplementary Fig. S6b). To extend these findings, spatial cell–cell interaction was conducted via stlearn (Supplementary Fig. S6c, d). The SLAMF7 signaling was detected to be upregulated among tumor cells, and further activated in invasive fronts and the metastasis spots at the benign side of invasive frontiers (Fig. 1k–l; Supplementary Fig. S6d). The spots distributed alongside the benign side of invasive fronts were active in LRP1-related signaling (Fig. 1l; Supplementary Fig. S6c). In keeping with that, the spots with high metastatic scores were dominantly found at the tumor side of the margins (Supplementary Fig. S7). In vitro, the expression of Igs and invasive ability of SCLC cell lines were further validated (Supplementary Fig. S8). Hence, cells at the tumor margin were characterized by the expression of SLAMF7 and LRP1, respectively. The distribution of SLAMF7, IGHA1, CD68, and LRP1 was confirmed by mIF (Fig. 1k). Moreover, in an SCLC cohort of our institution with bulk-RNA sequencing data, 78 patients were classified into four groups based on the expression of both ASCL1 and IGHA1 (ASCL1^–^Ig^–^, ASCL1^+^Ig^–^, ASCL1^–^Ig^+^, and ASCL1^+^Ig^+^) (Supplementary Fig. S9, Table S1). Our data suggested that the ASCL1^–^Ig^+^ group of patients (*n* = 32) had a significantly poor progression-free survival (PFS, *P* = 0.0053) and overall survival (OS, *P* = 0.011) compared with the ASCL1^+^Ig^–^ group (*n* = 8) (Fig. 1m, n).

In this study, we employed a combination of spatially resolved transcriptomics and multi-regional scRNA-seq to comprehensively characterize the transcriptional landscape of both SCLC tumor cells and the adjacent tumor microenvironment. A key finding of our study is the identification of a previously unrecognized subtype of SCLC cells characterized by the expression of Ig. Notably, these Ig-expressing SCLC cells were predominantly localized at the interface between the tumor and normal lung tissues, exhibiting invasive features. Our data reveal a spatially resolved transcriptomic heterogeneity within SCLC and provide mechanistic insights, as well as potential targets for therapeutic interventions in SCLC.

### Supplementary information

Supplementary information, Figures and Tables

## Data Availability

The scRNA-seq and spatial resolved transcriptomics datasets are available in the NCBI Sequence Read Archive repository with the following BioProject and SRA accession numbers: PRJNA903556, SRR22352803, SRR22352798, SRR22352800, SRR22352796, SRR22352795, SRR22352794, SRR22352793, SRR22352792, SRR22352799, SRR22352801, SRR22352797, SRR22352802, SRR22352791, SRR22352790, SRR22352789, SRR22352788, SRR22352787, and SRR22352786. The clinical information and bulk-RNA sequencing data of the lung cancer cohort in this article are available upon request.
