# Supplementary material for: Deciphering the role of immunoglobulin secreting malignant lineages in the invasive frontiers of small cell lung cancer by scRNA-seq and spatial transcriptomics analysis
Source: Cell Discov. 2023 Dec 12;9:123. doi: 10.1038/s41421-023-00621-4 (PMC10713609; doi:10.1038/s41421-023-00621-4)
Supplement: Supplementary file 1 — Supplementary information, Figures and Tables [file 41421_2023_621_MOESM1_ESM.pdf]

Supplementary Materials for

Deciphering the Role of Immunoglobulin Secreting Malignant Lineages in the Invasive Frontiers of Small Cell Lung Cancer by Single-cell RNA-sequencing and Spatial Transcriptomics Analysis

Fei Wu, PhD[1,2#], Xiao Zhang, PhD[1#], Minglei Wang, PhD[1], Jingxin Zhang, MASc[1], Minxin Chen, BSc[1], Ziyuan Ren, BSc[1], Meng Wu, PhD[1], Pingping Song, MD[3*], Jinming Yu, MD[1,4*], Dawei Chen, PhD[1*]

[1]Department of Radiation Oncology and Shandong Provincial Key Laboratory of Radiation Oncology, Shandong First Medical University and Shandong Academy of Medical Sciences, No. 440 Jiyan Rd., Jinan, Shandong, China.

[2]Department of Urology, Shandong Provincial Hospital Affiliated to Shandong First Medical University, No.325 Jingwu Rd., Jinan, Shandong, China

[3]Department of Thoracic Surgery, Shandong Cancer Hospital and Institute, Shandong First Medical University and Shandong Academy of Medical Sciences, No. 440 Jiyan Rd., Jinan, Shandong, China.

[4]Research Unit of Radiation Oncology, Chinese Academy of Medical Sciences, No. 440 Jiyan Rd., Jinan, Shandong, China.

[#] These authors contributed equally to this study;

[*]Correspondence should be addressed to:

Pingping Song (spp128@126.com)

Jinming Yu (sdyujinming@126.com)

Dawei Chen (dave0505@yeah.net)

[*]Corresponding authors' address: No.440 Jiyan Road, 250117, Jinan, Shandong Province, P.R. China, Tel: (+86)053187984777, Fax: (+86)053187984079, email: sdyujinming@126.com.

This PDF file includes:

Materials and Methods

Supplementary Figures S1 - S9

Supplementary Tables S1 - S3

Materials and Methods

Patient samples.

Clinical tissue samples were obtained from pulmonary lobectomy, pathologically diagnosed as small cell lung cancer (SCLC) by intra-operative frozen and permanent sections. Three SCLC patients were enrolled for high-throughput RNA sequencing that had not received antitumor therapy before surgery, including radiotherapy, chemotherapy, or immunotherapy. Fresh tumor margins, adjacent tissues, and distant normal lung tissues were obtained immediately after surgical resection and transferred to the 2ml freezing tube filled with Tissue Storage Solution (#130-100-008, Miltenyi Biotec GmbH, Bergisch Gladbach, Germany) and transported rapidly to the laboratory on ice. Notably, the adjacent normal tissues were 2 cm away from the matched tumor tissue. In addition, the distant normal tissues were 5 cm away from the matched tumor tissue. This study was conducted following the ethical standards of the Institution's Research Ethics Committee with patients' informed consent. Written informed consent was obtained from the three in this research for the usage of surgical samples as well as their clinical information.

Preparation of single-cell suspensions

Samples from each location of a single patient were divided into two pieces for single-cell RNA sequencing and spatial resolved transcriptomic sequencing, respectively. To prepare samples for single-cell RNA sequencing, one piece of tissue was cut into small pieces about 1 mm^3 in size and placed in a petri dish with an appropriate amount of ice-cold PBS. Specimens were washed twice with PBS and digested with Miltenyi Tumor Dissociation Kit (#130-095-929, Miltenyi Biotec GmbH) and the GentleMACS (#130-093-235, Miltenyi Biotec GmbH) following the manufacturer's instructions. The dissociated cells were subsequently passed through a 70 μm cell strainer (#08-771-2, Corning Falcon, USA) to remove clumps and undigested tissue. After centrifugation, the cell pellet was washed twice with PBS supplemented with 1% fetal bovine serum (#10100147, Gibco by Life Technologies, Carlsbad, CA, USA).

Libraries construction and single-cell RNA sequencing

Single-cell RNA sequencing (scRNA-seq) libraries were generated using the Chromium Single Cell 3′ library and Gel Bead & Multiplex Kit (a technology based on the 10X Genomics platform, 10X Technologies, Inc. Pleasanton, CA, USA) in compliance with the manufacturer's recommendations, which could isolate and label 5000 - 10000 single cells at a time. In brief, the gel beads with bar codes and single cells were wrapped in oil droplets. After the gel bead dissolves within each oil droplet, the cell cleaves to release mRNA, reverse transcribed to produce barcoded cDNA for sequencing. Multiple 10x Genomics cDNA libraries were generated from distinct samples to account for different types of injury. All libraries were sequenced on an Illumina NovaSeq 6000 until sufficient saturation was reached. Reads with less than 30 were filtered out, and CellRanger (10x Genomics) was used to align reads onto the mm10 reference genome and gene expression quantification.

Preparation for spatial resolved transcriptomic sequencing

To prepare samples for spatial resolved transcriptomic sequencing. Fresh lung tissues or tumor samples were embedded and cryosectioned on dry ice. The cryosections were collected in enzyme-free tubes for RNA extraction, and the RIN value of RNA was more significant than 7. Since the optimal permeabilization time for samples from different locations and tissue type is different, tissue optimization was performed before spatial transcriptome sequencing experiments. Frozen sections were fixed on the Slide Cassette from a Visium Slide kit (10X Genomics), and tissue permeabilization was performed after H&E staining and imaging. A permeabilization enzyme was applied to permeabilize the lung tissues on the slide for incubation for the predetermined duration (20min, 24min, 30min). Under tissue permease, the polyadenylated mRNA released by the cells was captured by a probe fixed on the chip to form fluorescent-labeled cDNA. The optimal permeabilization time was 30 min, determined by fluorescence imaging results in our pilot study. After washing by 0.0.1× saline sodium citrate buffer (#S0902, Millipore Sigma, Darmstadt, Germany), RT Master Mix (Visium Reagent kit, 10X Genomics) containing reverse transcription reagents was added to the permeabilized tissue sections in the Thermocycler Adaptor. Incubation with the reagents produces spatially barcoded full-length cDNA from polyadenylated mRNA on the slide. The libraries' construction and

sequencing were the same as described above. After the next-generation sequencing, raw FASTQ files of 10X Visium data and histology images were processed with the Space Ranger software (STAR v.2.5.1b) for genome alignment against the Cell Ranger hg38 reference genome (GRCh38, http://cf.10xgenomics.com/supp/cell-exp/refdata-cellranger-GRCh38-3.0.0.tar.gz).

Quality control of scRNA-seq data

Cell expression profiles were quantile normalized and analyzed using the Seurat (http://satijalab.org/seurat/). The standard Seurat workflow was performed with the pooled scRNA-seq data from 10X Genomics using the R software (version 4.1.1) package Seurat (v4.05). Briefly, quality control before analysis on each case was performed on 'nFeature_RNA', 'nCount_RNA', 'percent_mt', 'percent_ribo', 'percent_hb' in each cell. For each case, the total number of genes detected per cell (nFeature_RNA), number of transcripts per cell (nCount_RNA), and percentage of transcripts mapping to mitochondrial genes ('percent_mt'), ribosome genes (percent_ribo), and hemoglobin genes (percent_hb) were visualized (Supplementary Fig.S1A). Samples with less than 3 cells and/or less than 300 detected genes per cell, as well as percent_mito < 5, percent_ribo > 3 and percent_hb < 0.1, were excluded from further analysis. Doublets were identified and filtered by R package DoubletFinder (v3), with 4% doublets expected (Supplementary Fig.S1C). Then we used Seurat (v4) to detect highly variable genes, perform PCA, graph-based clustering, t-SNE, and Uniform Manifold Approximation and Projection (UMAP).

Integration and clustering

Integration anchors were identified in the list of samples using the Canonical Correlation Analysis (CCA) method to integrate multiple samples. For clustering, principal component analysis (PCA), T-distributed Stochastic Neighbor Embedding (t-SNE), and Uniform Manifold Approximation and Projection (UMAP) were performed for dimension reduction. Furthermore, marker genes of each respective cluster were identified by the function of 'FindAllMarkers' and the package of 'SingleR' previous to manual annotation. In addition, the expression of the top 20 differentially expressed marker genes was manually checked in the literature and separated for further analysis. Where a cluster could not be identified using known marker genes, they were identified by a highly discriminative gene that was among the most differentially expressed in that cluster. To obtain the most sensitive and specific differentially expressed genes for subpopulations, we identified genes with a *P*-value less than 10^{-5} and an average log fold-change more significant than 2.

Processing, integration, and visualization of 10X Visium data

Spatial resolved scRNA-seq data from four 10x Visium capture areas was normalized using sc-transform function of the Seurat V4.06 package and then used for batch correction[1]. The updated Seurat (v4.06) package was applied to select top variable genes for spatial scRNA-seq clustering. In brief, the FindVariableFeatures function was used to choose the top 2000 highly variable genes from the data. Mean centering and scaling, followed by principal component analysis on a matrix composed of spots and gene expression counts, were conducted for the reduction of dimensions according to the Seurat pipelines for 10X visium (https://satijalab.org/seurat/articles/spatial_vignette.html#slide-seq-1). UMAP was initialized in this PCA space to visualize the data on reduced UMAP dimensions. The spots were clustered on PCA space using the Shared Nearest Neighbor (SNN) algorithm implemented as FindNeighbors and FindClusters in Seurat v4 with dim 1:30. The spot clusters were then visualized on UMAP space using the SpatialDimPlot function. The FindAllMarkers function in Seurat with its default parameters was used to get a list of differentially expressed genes for further annotation. The expression of indicated genes was visualized with the SpatialFeaturePlot function. In addition, we also analyzed cell-cell communication and inference of spatial trajectory by integrated analysis of spatial information and transcriptomic data using the stlearn (Version 0.4.9, https://github.com/BiomedicalMachineLearning/stLearn). Metastasis score was evaluated by the AddModuleScore function of Seurat with the average expression of 66 genes associated with the metastasis of solid tumor based on the Gene Set Enrichment Analysis (GSEA) database (https://www.gsea-msigdb.org/gsea/msigdb).

Analysis of sub-clusters

Malignant epithelial cells were extracted via the 'SubsetData' function following primary annotation. Then, the 'FindClusters' and 'FindAllMarker' functions were conducted, and the malignant epithelial cells were re-clustered by tSNE and UMAP. The sub-clusters were annotated by the dominant expression cell markers. The following cutoff threshold values were applied to reveal the marker genes for each cluster: adjust *P*-value < 0.01, and fold change > 0.5.

Cellular communications analysis

To understand the global communications among cells, a Cellchat algorithm was applied to quantitatively infer and analyze intercellular communication networks from scRNA-seq data (http://www.cellchat.org/)[2]. The Cellchat algorithm uses a database of interactions among ligands, receptors, and their cofactors that accurately represent known heteromeric molecular complexes. In this study, default settings were used as the Cellchat algorithm recommended. To identify significant differential interactions between the iRT and control groups, we performed the differential analysis functions of Cellchat ('compareInteractions', 'netVisual_diffInteraction'). The cell-cell interactions were visualized with 'circlize' and 'pheatmap'.

Scenic transcriptional factor analysis

Single-cell regulatory network inference and clustering (SCENIC) analysis was performed on different groups of samples using the 'SCENIC' package[3]. SCENIC can identify translational factors (TFs) targets based on the mRNA expression network. In addition, SCENIC was used to perform TFs motif enrichment analysis to identify direct targets and score the activity of the regulators (AUCell algorithm). The activity TFs were visualized in a heatmap, and specific TFs were visualized in a dot plot.

Analysis of copy number variations

The gene expression data of epithelial cells extracted from the Seurat object were analyzed for the copy number variations (CNVs) with the inferCNV program of the Trinity CTAT Project (https://github.com/broadinstitute/inferCNV) and CopyKAT (https://github.com/navinlabcode/copykat)[4, 5]. As malignant cells harbor significantly more CNVs than normal cells, we estimated CNVs from scRNA-seq following the steps described in the previous study and made some minor improvements[6]. The transcriptomic profiles of the natural killer (NK) cells from the lung tissues were set as reference cells. Among the different groups for comparison, 5000 cells from each group were randomly sampled after quality control filtering with greater than 3000 UMIs. Each CNV was annotated to be either a gain or a loss.

The high dimensional weighted gene co-expression network analysis

The high dimensional weighted gene co-expression network analysis (hdWGCNA) algorithm was used in this study to find the various expressed modules among immunoglobin-high and immunoglobin-low cancer cells with high biological significance and investigate the association between gene networks and diseases (https://github.com/smorabit/hdWGCNA)[7]. The module eigengene (M.E.) was defined to summarize the expression profiles of each module. Furthermore, the significance of the correlation between M.E.s and lesion types were calculated.

Measuring gene activity in individual cells

The raw data from scRNA-seq were aligned using the Cell Ranger pipelines and were sorted by using the updated SAMtools[8]. Then, the spliced and un-spliced reads were counted using velocyto (python version), and generate loom files for each sample[9]. Gene-specific velocities were then analyzed via scVelo package[10]. Moreover, plots for the ratio between un-spliced and spliced mRNA, for the velocity and the expression of various individual genes were obtained using scVelo with the velocity information. Following the pipeline in scVelo (https://scvelo.readthedocs.io/), the same projection of UMAP was generated by setting the default parameters.

Gene set variation analysis

To estimate the variation of pathway activity over different cell populations in an unsupervised manner, the method of Gene Set Variation Analysis (GSVA) was applied in this study. Unlike GSEA, GSVA does not

require differential analysis previously. The GSVA algorithm calculates the variation score of a specific set of genes in subpopulations of cells based on the expression matrix. In this study, the output of the GSVA calculation was a variation score matrix. To be more objective in estimating the difference, AUCell, UCell, singlescore, ssgsea, and RRA were used simultaneously to score the variation of the indicated subpopulation.

Gene set enrichment analysis

Gene set enrichment analysis (GSEA) (http://software.broadinstitute.org) was conducted between the control group and the treated group to explore the potential mechanisms involved in the pathogenesis of the treated [11, 12]. The enrichment analysis was performed using the Molecular Signatures Database (MSigDB) of c5. The enriched gene sets in the GSEA that reached a nominal significance level of $P < 0.05$ were considered significant.

Differentially expressed gene analysis

The 'Limma' package was used to perform the analysis for the differentially expressed gene (DEG). An empirical Bayesian method was conducted to estimate the fold change between immunoglobin-high and immunoglobin-low cancer cells. The adjusted *P*-value for multiple testing was calculated using the Benjamini–Hochberg correction. The genes with an absolute $\log_2$ fold change greater than two were identified as DEGs between the two groups.

The SCLC cohort

An advanced small cell lung cancer (SCLC) cohort with bulk RNA sequencing data from our institution was used. The cohort included 78 patients diagnosed with SCLC and treated with first or second-line chemotherapy or radiotherapy at Shandong Tumor Hospital. Clinical data were collected by Mrs. J.Z., Miss. M.C. and Mr.Z.R.. All the clinical information and paraffin sections were collected, stored, and used with the written informed consent of the patients, and bulk-RNAseq data was generated with the next-generation sequencing. Written informed consent was obtained from all patients before enrolment. The hospital institutional review board approved the study in accordance with the principles of the Declaration of Helsinki and applicable local regulations.

Immunohistochemistry

Tumor tissues from three SCLC patients were provided by the Department of Pathology, The Shandong Cancer Hospital, and Shandong First Medical University. In brief, from each corresponding paraffin block, two 2.0-mm cores were made. All the samples collected before targeted therapy or immunotherapy were stained with antibodies against ASCL1 (1:400 dilution, # ab211327, Abcam, MA, United States), IGHA (1:400 dilution, #GTX60873, GeneTex, United States), and KI67 (1:400 dilution, #ab15580, Abcam). The protocol for immunohistochemistry (IHC) staining was described as previously[13]. The universal secondary protocol and the 3,3'-Diaminobenzidine (DAB, ZLI-9019, ZSGB-Bio, Beijing, China) were used to amplify the signaling.

Multiplexed immunofluorescence staining

To further explore the co-expression of senescence markers, multiplexed immunofluorescence (mIF) staining was conducted with fluorescent tyramine signal amplification reagents14. In brief, the paraffin blocks (4 blocks in each treatment group) were sectioned for multiplexed immunohistochemical staining with three μm thickness, followed by deparaffinized with xylene and gradient ethanol solutions and antigen retrieval. Next, in order to block the binding of unspecific antibodies, opal antibody diluent/block (Akoya Biosciences, MA, United States) was used. For multiplex immunofluorescence staining, we followed the Opal protocol staining method for the following markers: anti-CD20 (dilution 1:200, # ab78237, Abcam) was labeled with Akoya Opal fluorophores 520; anti-SLAMF7 (dilution 1:200, #98611S, Cell Signaling Technology, MA, USA) were labeled with Akoya Opal fluorophores 480; anti-LRP1 (dilution 1:200, #ab92544, Abcam) was labeled with Akoya Opal fluorophores 520; anti-KI67 (1:200 dilution, #ab15580, Abcam, MA, United States) was labeled with Akoya Opal fluorophores 570; anti-IGHA (1:200 dilution, #GTX60873, GeneTex) was labeled with Akoya Opal fluorophores 620; anti-CD68 (dilution 1:200, #ab213363, Abcam) was labeled with Akoya Opal fluorophores 780; nucleus were labeled with DAPI (1:100, Akoya Biosciences). All sections were cover-slipped using an Anti-Fade Fluorescence Mounting Medium (Ab104135, Abcam). Whole slide tissue scanning

was performed at 40× magnification using the Vectra Polaris System (Akoya Biosciences). The identification of positive cells was analyzed with the open-source software QuPath using a custom, unsupervised algorithm[15].

Cell culture

The human SCLC cell lines DMS53, NCI-H146, NCI-H524, NCI-H526, and SW1271 were obtained from American Type Culture Collection (ATCC), all cell lines were authenticated by short tandem repeat (STR) profiling and has been tested for mycoplasma contamination. SW1271 cells were maintained in Dulbecco's modified Eagle's medium (DMEM) basic medium supplemented with 10% Fetal Bovine Serum (FBS; Gibco), 100 U/mL penicillin, and 100 μg/mL streptomycin, while others were were cultured in RPMI1640 and supplemented with the aforementioned components.

Quantitative and reverse transcriptional PCR

Total RNA from SCLC cells was extracted using TRIzoL reagent (Invitrogen). A total of 1μg RNA was reverse transcribed into cDNA. Subsequently, qPCR was performed employing HiScript® SuperMix for qPCR (+gDNA wiper) system in according to the manufacturer's instruction. The expression of ASCL1, IGKC, IGLC2, IGHG3 and IGHA1 was normalized to glyceraldehyde-3-phosphate dehydrogenase (GAPDH) and determined as $2^{-\Delta CT}$. All primers were synthesized by Accurate Biotechnology (Hunan) Co.,Ltd and are listed in Supplementary Table S2.

Cell migration and invasion assays

The invasion and migration abilities of SCLC cells were carried out by Transwell Chambers (8-μm pore size; Corning Costar, Cambridge, MA, USA). For invasion assays，the upper chambers were pre-coated with 50 μL diluted Matrigel (1:5, BD Biosciences, Bedford, MA, USA). Briefly, 2 x 10^4 SCLC cells in 500 μL serum-free medium were seeded in upper chambers, and 700 μL medium with 20% FBS was added into lower chambers. After the appropriate times (monolayer cells for 48h and suspension cells for 72h), the non-invading cells on the upper surface of the membrane were removed and the invading cells were fixed with 4% paraformaldehyde for 30 min and subsequently stained with 1% crystal violet for 30 min. After that, the numbers of cells were caculated through randomly selecting three fields of view at 100 magnification.

Western blot assay

The SCLC cells were harvest and lysed with RIPA lysis buffer to extract proteins as previous study. For non-denatured and non-reducing protein, cells were lysed with non-denatured RIPA tissue/cell lysate (Solarbio, Beijing, China) and mixed with sample loading buffer (Beyotime, Beijing, China). After that, the proteins were separated on 10% BeyoGel™ Plus PAGE Precast Gel (Beyotim) using non-denatured PAGE electrophoresis solution (Beyotime). Under non-denatured and non-reducing conditions, all reagents do not contain SDS, DTT and 2-Mercaptoethanol, and are not denatured by boiling at 10 ℃. Subsequently, the proteins were transferred into polyvinylidene difluoride (PVDF) membrane and blocked with 5% skim milk for 1 h. Then, the protein-loaded PVDF membrane was incubated with the primary antibodies overnight at 4 °C. After that, the PVDF membrane was incubated with the secondary antibody at room temperature for 1 h. Finally, the protein band was developed by enhanced chemiluminescence (ECL). All antibodies and their dilutions are listed in Supplementary Table S3.

Statistical analysis

Statistical analysis was performed with the R (v3.6.1), SPSS (v22, IBM, Armonk, NY), and Prism 6.0 (SanDiego, CA) software. Comparisons were performed using $\chi2$ test and unpaired two-sided Wilcoxon rank-sum test unless specified. The cumulative survival time was estimated by Kaplan–Meier estimator with a log-rank test.

References

1 Hafemeister C, Satija R. Normalization and variance stabilization of single-cell RNA-seq data using regularized negative binomial regression. *Genome Biol*. **20**:296(2019).

2 Jin S *et al.* Inference and analysis of cell-cell communication using CellChat. *Nat Commun*. **12**:1088(2021).

3 Aibar S *et al.* SCENIC: single-cell regulatory network inference and clustering. *Nat Methods*. **14**:1083-1086(2017).

4 Gao R *et al.* Delineating copy number and clonal substructure in human tumors from single-cell transcriptomes. *Nat Biotechnol*. **39**:599-608(2021).

5 Patel AP *et al.* Single-cell RNA-seq highlights intratumoral heterogeneity in primary glioblastoma. *Science*. **344**:1396-1401(2014).

6 Venteicher AS *et al.* Decoupling genetics, lineages, and microenvironment in IDH-mutant gliomas by single-cell RNA-seq. *Science*. **355**(2017).

7 Morabito S *et al.* Single-nucleus chromatin accessibility and transcriptomic characterization of Alzheimer's disease. *Nat Genet*. **53**:1143-1155(2021).

8 Li H *et al.* The Sequence Alignment/Map format and SAMtools. *Bioinformatics*. **25**:2078-2079(2009).

9 La Manno G *et al.* RNA velocity of single cells. *Nature*. **560**:494-498(2018).

10 Bergen V *et al.* Generalizing RNA velocity to transient cell states through dynamical modeling. *Nat Biotechnol*. **38**:1408-1414(2020).

11 Subramanian A *et al.* Gene set enrichment analysis: a knowledge-based approach for interpreting genome-wide expression profiles. *Proc Natl Acad Sci U S A*. **102**:15545-15550(2005).

12 Mootha VK *et al.* PGC-1alpha-responsive genes involved in oxidative phosphorylation are coordinately downregulated in human diabetes. *Nat Genet*. **34**:267-273(2003).

13 Fragkoulis C *et al.* Expression of proto-oncogene c-Myc in patients with urinary bladder transitional cell carcinoma. *Curr Urol*. **15**:231-233(2021).

14 Zhang W *et al.* Fully automated 5-plex fluorescent immunohistochemistry with tyramide signal amplification and same species antibodies. *Lab Invest*. **97**:873-885(2017).

15 Bankhead P *et al.* QuPath: Open source software for digital pathology image analysis. *Sci Rep*. **7**:16878(2017).

Supplementary Figures

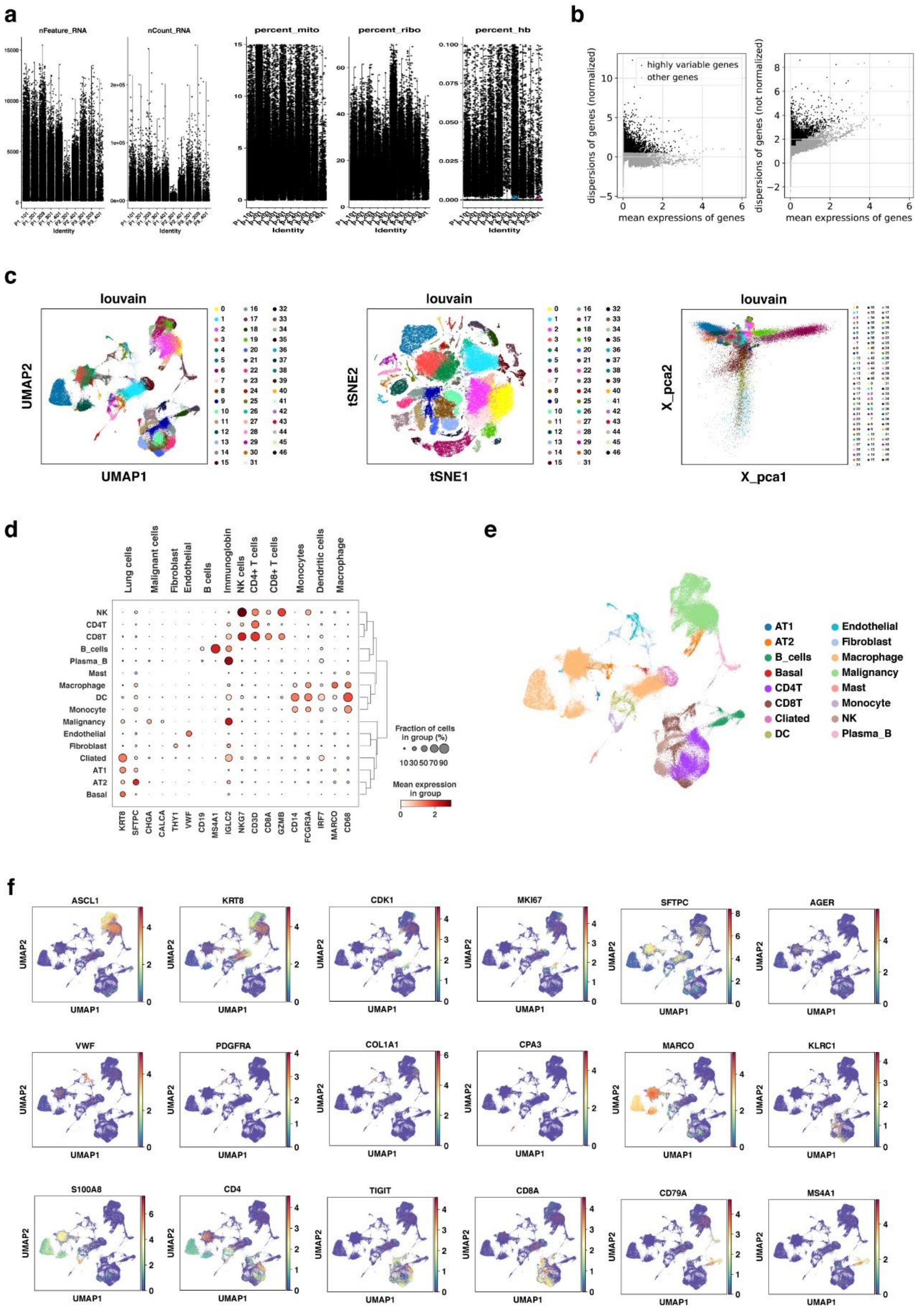

Supplementary Figure S1 The quality control process and annotation of single-cell RNA sequencing data of small cell lung cancer. (**a**) The percentage of "nFeature_RNA", "nColunt_RNA", "percent_mito" (mitochondria genes), "percent_ribo" (ribosome genes), and "percent_hb" (hemoglobin genes) after the filtration of genes under indicated cutoff values. (**b**) The expression of highly variable genes was visualized in scatter plots before and after normalization. (**c**) Uniform Manifold Approximation and Projection (UMAP), t-distributed stochastic neighbor embedding (tSNE), and principal component analysis (PCA) embedding of jointly analyzed single-cell transcriptomes from 109,462 cells from small cell lung cancer (SCLC) tumors. (**d**)

Distribution of canonical molecular markers for indicated subpopulations visualized in the dot plot. (**e**) UMAP plot of Louvain clustering (Scanpy) of jointly analyzed single-cell transcriptomes grouped by 16 cell types. (**f**) Distribution of canonical molecular markers for indicated subpopulations visualized in the feature plot.

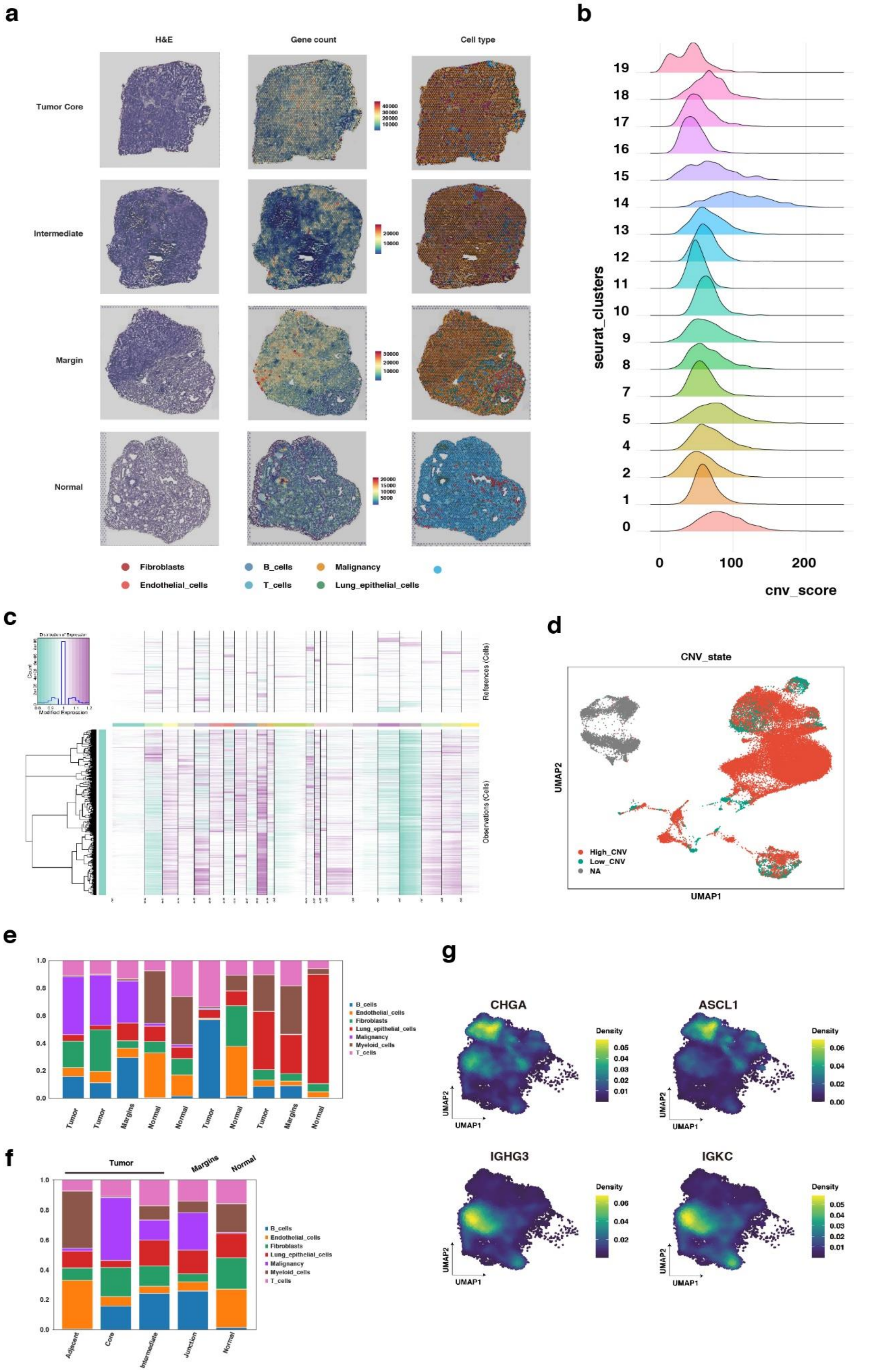

Supplementary Figure S2 The identification of malignant epithelial cells by copy number variations. (a) Hemoxylin and eosin (H&E) staining, gene count maps, and cell type maps of different sites of the SCLC

patient. (b) The CNV score of various clusters in epithelial cells from the SCLC was visualized in ridge plots. Malignant cells are characterized and identified by high CNV scores. (c) The estimation of copy number variants by inferCNV in malignant clusters with the reference of copy number variations from natural killer cells. (d) Uniform Manifold Approximation and Projection (UMAP) plot of epithelial and natural killer cells grouped by levels of CNV scores. (e-f) Cell proportion in each cell type grouped by different sample identities (e) or the sampling regions (f). (g) Expression of selected Ig genes (IGKC and IGHG3) and neuroendocrine markers (ASCL1 and CHGA) among malignant cells visualized in density plot.

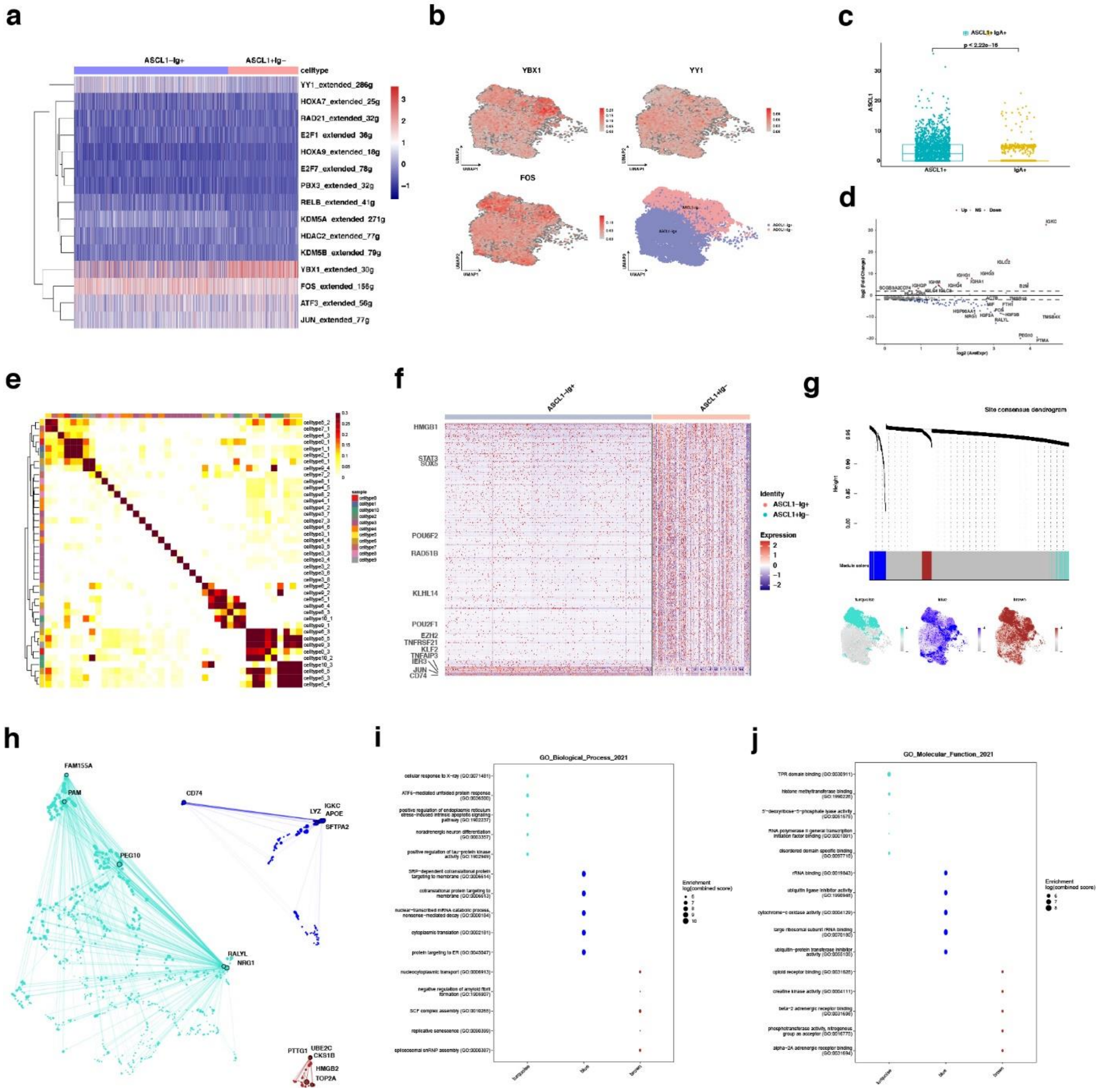

Supplementary Figure S3 The difference in gene expression and ontology between ASCL1^{+}Ig^{-} or ASCL1^{-}Ig^{+} cells. (**a**) A regulon activity heatmap of different malignant cells grouped by the expression of ASCL1 and Ig. The clustered regulon activity matrix reveals that the known cell types have distinct regulatory networks. (b) UMAP embedding of activity of indicated regulons (YBX1, YY1, and FOS) in malignant cells from SCLC patients. (c)Expression of ASCL1 in malignant cells grouped by the expression of ASCL1 and Ig based on Seurat clustering and feature plot visualization. (d) The volcano plot showed significantly upregulated genes (red) and down-regulated genes (blue) derived from a comparison between ASCL1 high and Ig high groups. (e) Hierarchical clustering on the Pearson correlation values of all malignant clusters analyzed by Consensus Non-negative Matrix factorization (cNMF, v1.4). (f) The heatmap showed significantly upregulated genes (red) and down-regulated genes (blue) derived from a comparison between ASCL1 high and Ig high groups. (g) With high-dimensional weighted gene co-expression network analysis

(hdWGCNA), three modules were identified in all the malignant cells. The expression of indicated modules was visualized in Uniform Manifold Approximation and Projection (UMAP) plot. (h) Modules were identified by performing Modularity Maximization on the network obtained from hdWGCNA. (i-j) The gene ontology (GO) analyses of significant module genes based on the GO biological functions database (i) and GO molecular functions database (j).

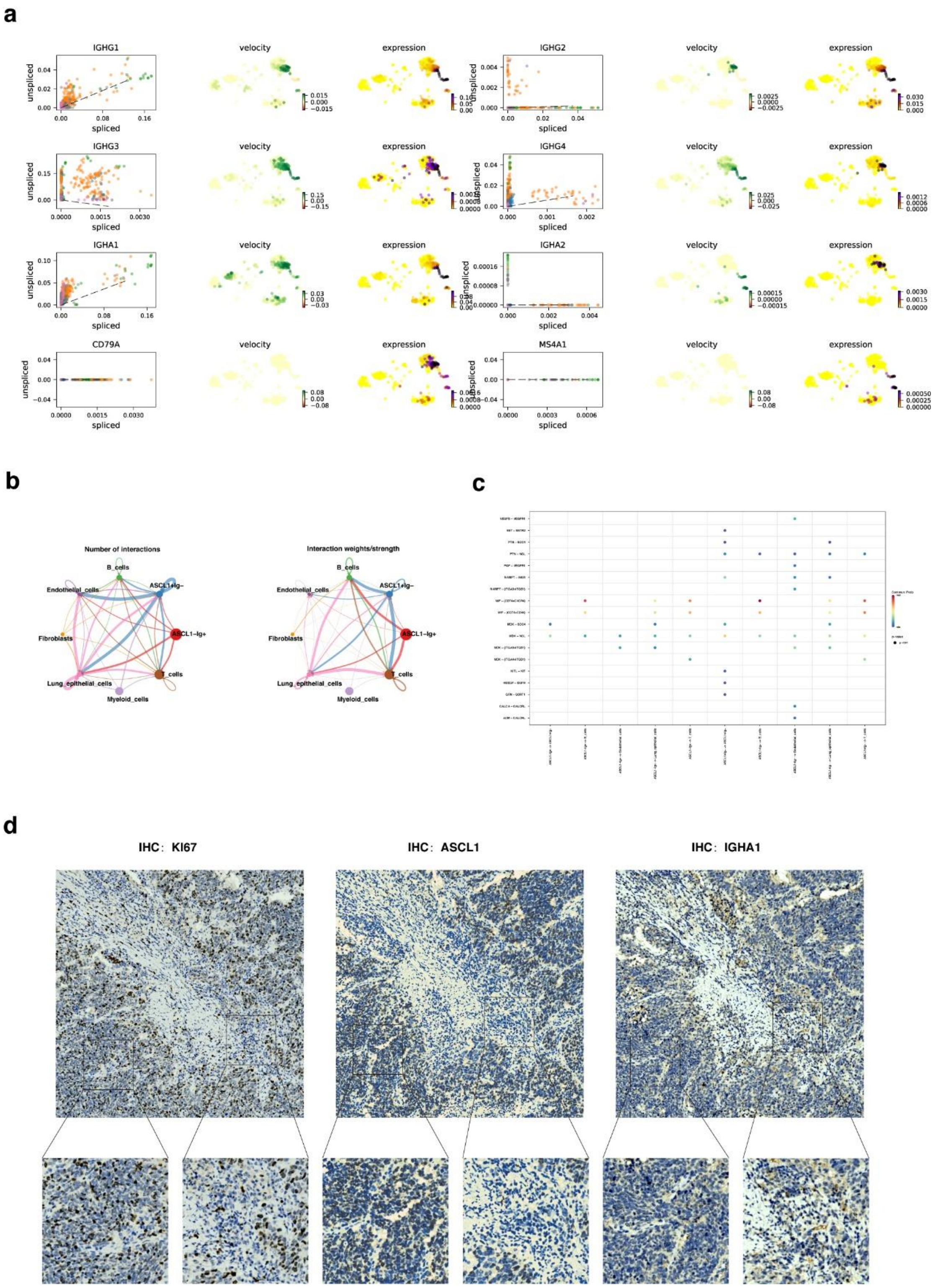

Supplementary Figure S4 The difference in RNA velocity between ASCL1^{+}Ig^{-} or ASCL1^{-}Ig^{+} cells. (a) A selection of phase portraits that show genes underlying the observed velocity field. RNA velocity unveils the dynamics of mRNA production. (b) Network view of the differential number of interactions among indicated

cell types. The ASCL1+Ig- and ASCL1-Ig+ cells have distinct cellular communication modes. (c) Differential activity of cell-to-cell signals derived from ASCL1+Ig- or ASCL1-Ig+ cells. (d) Representative image of the immunohistochemistry staining of KI67, ASCL1, and IGHA on serial sections from patients with small cell lung cancer.

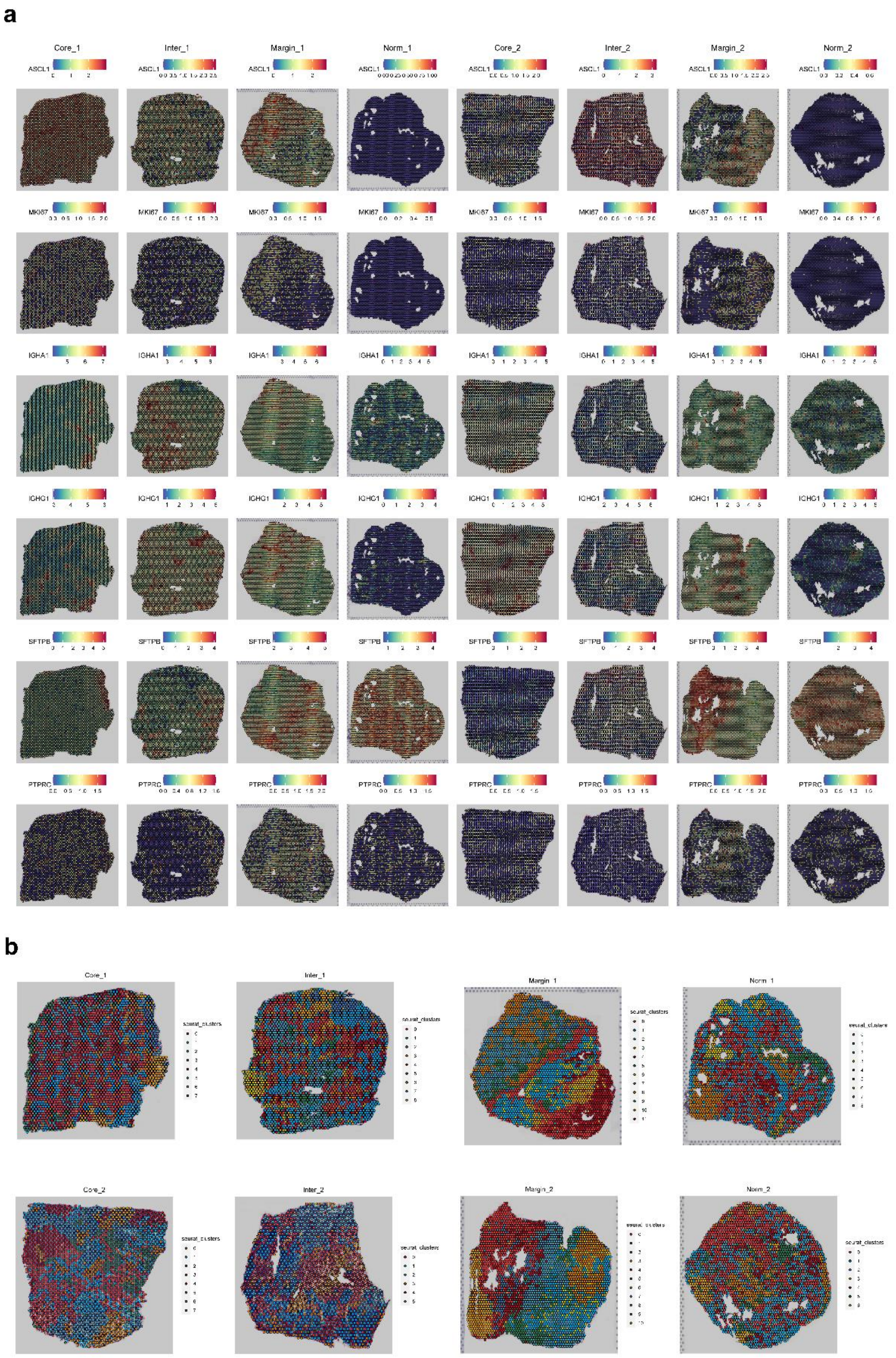

Supplementary Figure S5 Expression of marker genes and annotation of spots in 10X Visium slides. (a) Distribution of canonical molecular markers for neuroendocrine cells, malignant cells, immunoglobin, and alveolar epithelial cells in all the 10X Visium slides visualized in the spatially resolved feature plot. (b)

Annotation of dominant cell types in spots from the eight 10X Visium slides with canonical molecular markers.

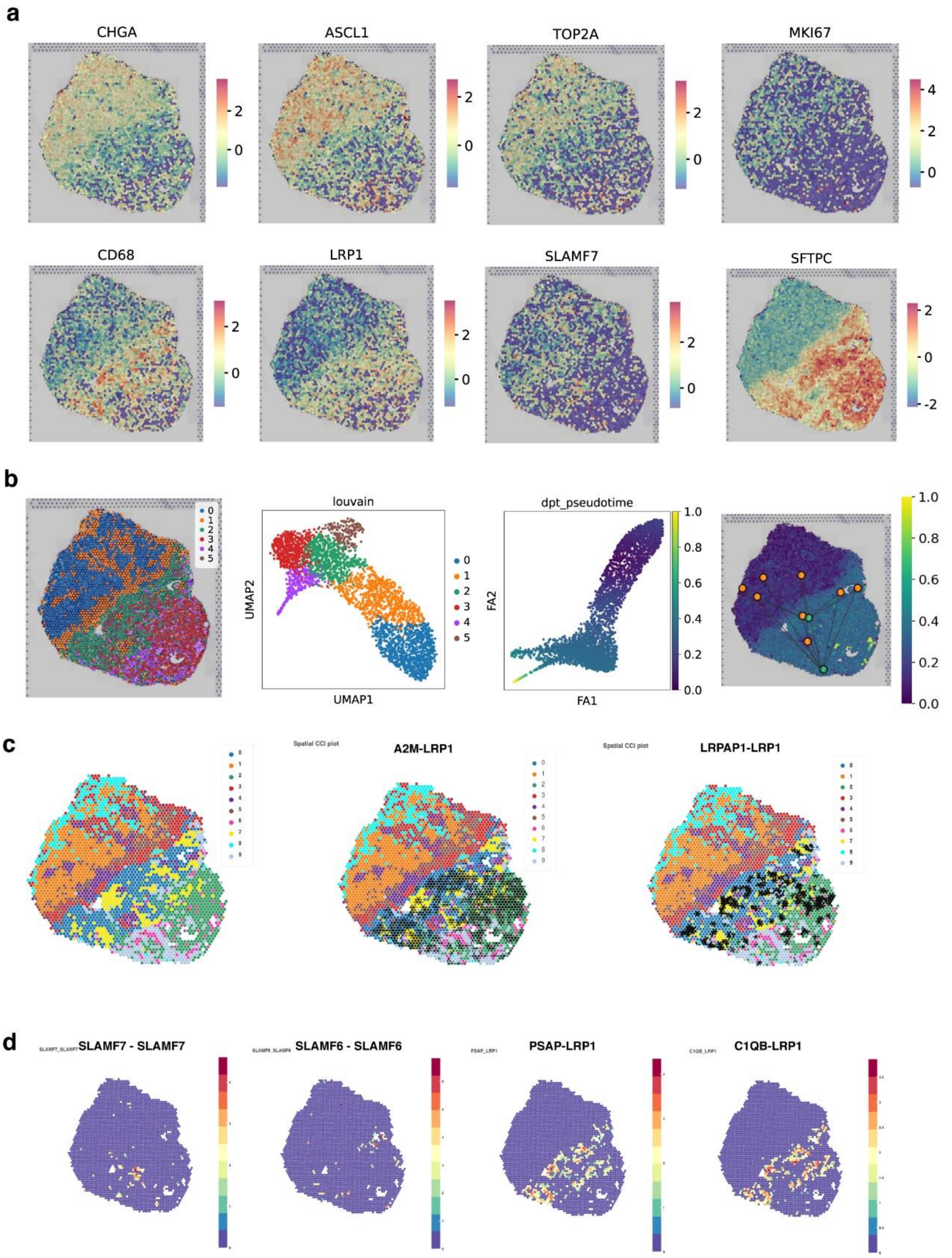

Supplementary Figure S6 Spatial cell-cell interaction analysis at invasive frontiers. (a) Expression of indicated genes (CHGA, ASCL1, TOP2A, MKI67, CD68, LRP1, SLAMF7, and SFTPC) for a 10X Visium slide with invasive frontiers from small cell lung cancer (SCLC). (b) Spatial trajectory inference analysis of cluster 1 spots using stlearn algorithm showing the intratumor heterogeneity of SCLC tumor cells with spatial information. (c) Spatial cell-cell interaction analysis of invasive frontiers of an SCLC patient using stlearn. Representative receptor and ligand pairs (A2M-LRP1 and LRPAP1-LRP1) are visualized. (d) Ligand receptor plot visualizing representative receptor and ligand pairs (SLAMF7-SLAMF7, SLAMF6-SLAMF6, PSAP-LRP1, and C1QB-LRP1) in a 10X Visium slides with invasive frontiers of SCLC.

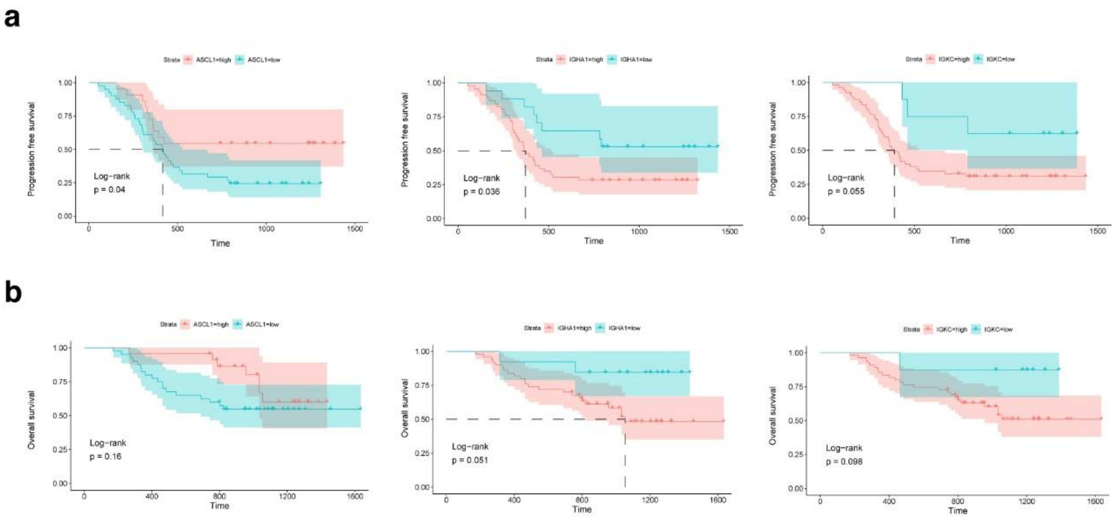

Supplementary Figure S7 The survival probability of small cell lung cancer patients grouped by ASCL1 or Ig expression. (a) Progression-free survival (PFS) curves of 63 patients with bulk-RNA sequencing information from our small cell lung cancer (SCLC) cohort, grouped by mRNA expression of ASCL1, IGHA1, or IGKC. (b) Overall survival (OS) curves of 63 patients with bulk-RNA sequencing information from our small cell lung cancer (SCLC) cohort, grouped by mRNA expression of ASCL1, IGHA1, or IGKC.

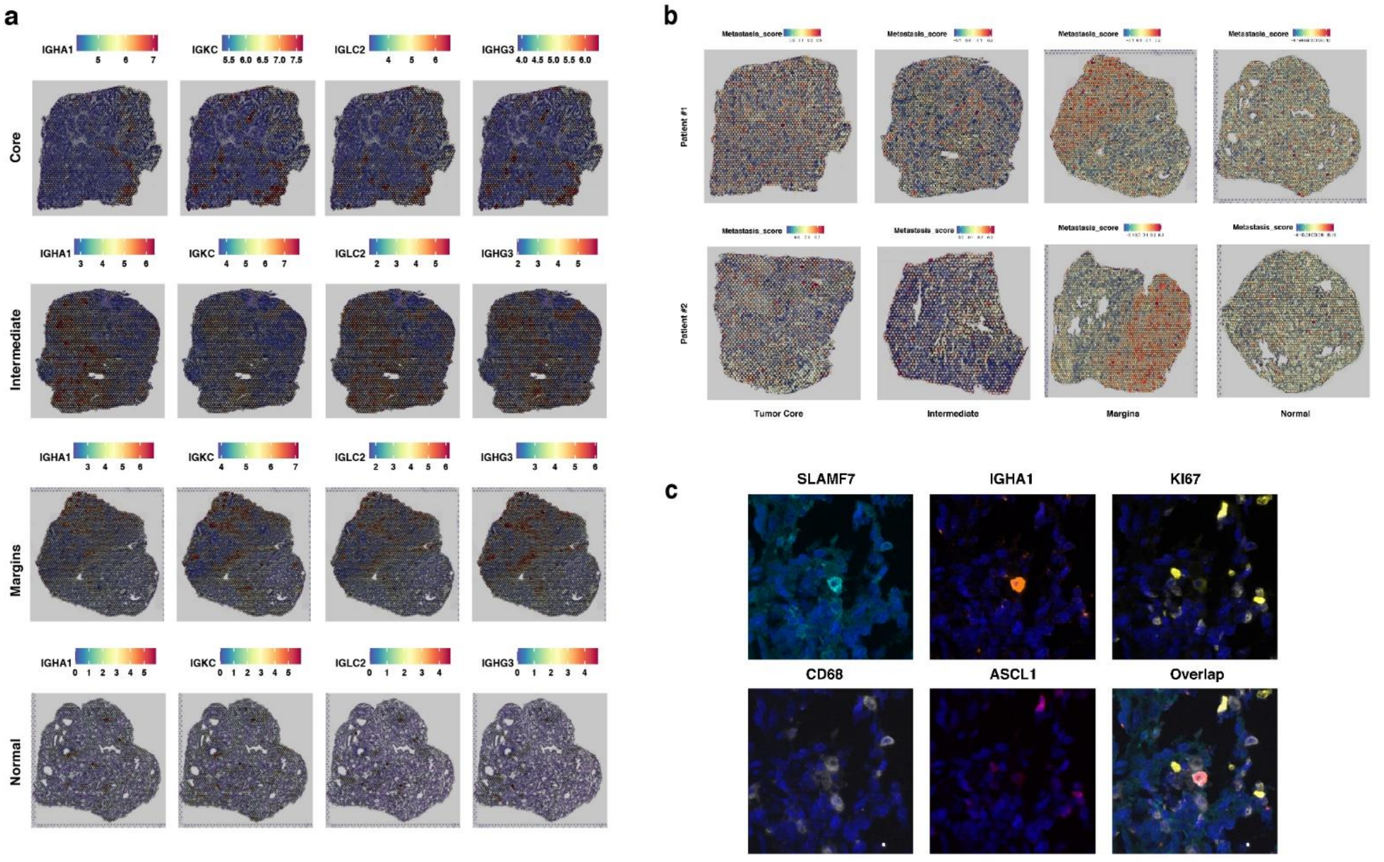

Supplementary Figure S8 The metastasis score analysis in the spatial resolved transcriptomic data. (a) Expression of selected Ig genes (IGHA, IGKC, IGLC2, and IGHG3) among spatial resolved transcriptomic data. (b) Using the average expression levels of these 66 genes upregulated in metastasis (GSEA, RAMASWAMY_METASTASIS_UP), we scored and visualized each spot on the spatial transcriptome. (c) Multiplexed IF staining (ASCL1, CD68, IGHA1, SLAMF7, KI67, and DAPI) showing co-aggregation of SLAMF7-high and IGHA1-high tumor cells (SLAMF7+IGHA1+ cells) in SCLC patients.

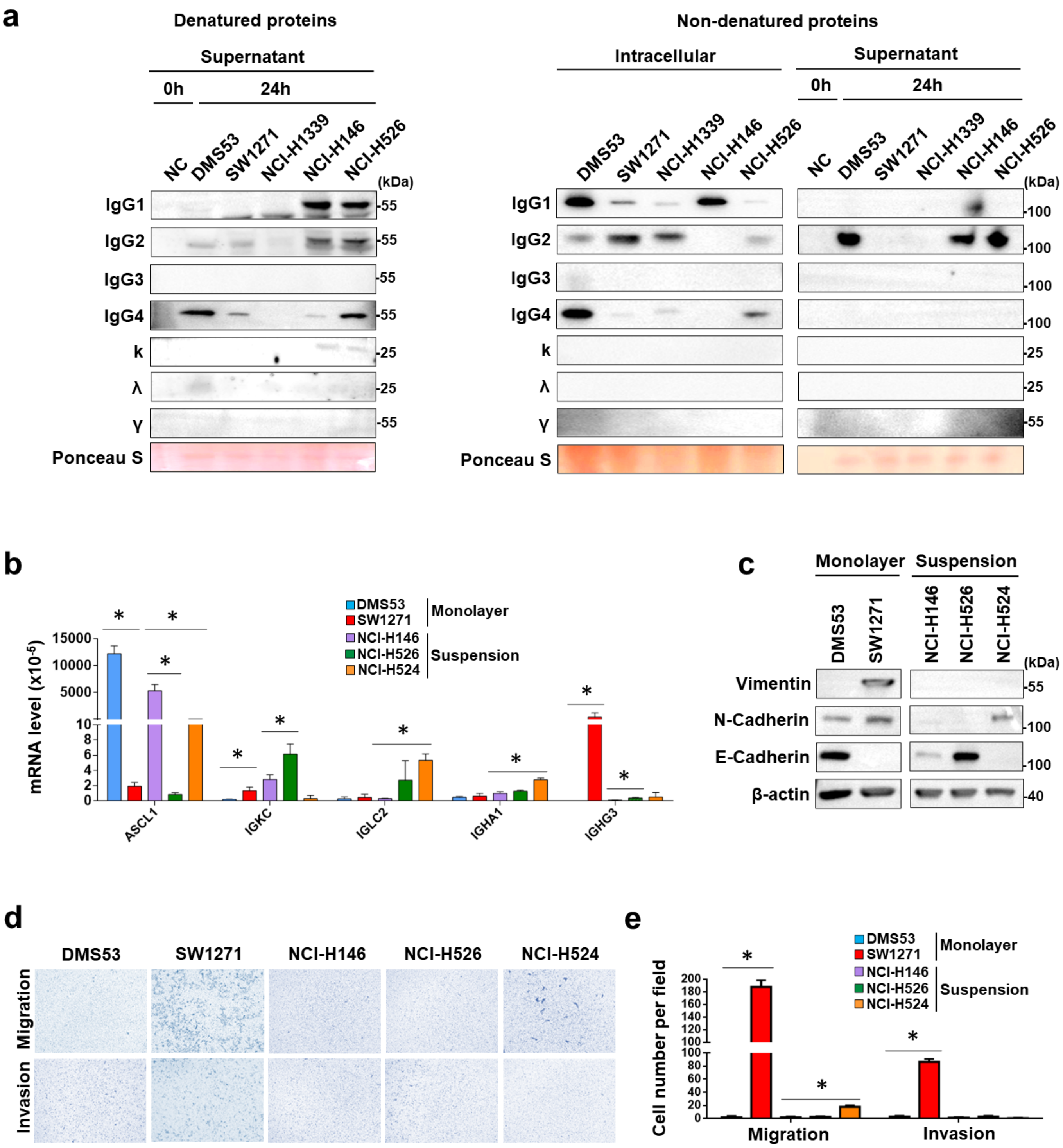

Supplementary Figure S9 The Ig expression was validated in multiple cell lines derived from small cell lung cancer.

(a) The expression pattern of IgG1, IgG2, IgG3, IgG4, free light chains κ, λ, and heavy chains γ in SCLC cell lines (DMS53, SW1271, H1339, NCI-H146, NCI-H526) was detected by western blot under denatured or non-denatured protein electrophoresis conditions. (b) The mRNA expression levels of ASCL1 and Ig genes (IGKC, IGLC2, IGHA1, IGHG3) were assessed by RT-qPCR. (c) The EMT markers were detected by western blot. (d) The migration and invasion ability of SCLC cells was detected by transwell assay.NCI-H146, NCI-H526) was detected by western blot under denatured or non-denatured protein electrophoresis conditions. (e) The migration and invasion ability of SCLC cells was statistically analyzed and visualized in bar plot.

Supplementary Table S1 Baseline characteristics of the small cell lung cancer cohort

	T1 (N=9)	T2 (N=35)	T3 (N=14)	T4 (N=20)	Overall (N=78)
**Sex**					
Female	3 (33.3%)	13 (37.1%)	3 (21.4%)	3 (15.0%)	22 (28.2%)
Male	6 (66.7%)	22 (62.9%)	11 (78.6%)	17 (85.0%)	56 (71.8%)
**Age**					
Mean (SD)	61.0 (10.0)	61.6 (7.96)	57.4 (7.62)	59.4 (8.93)	60.2 (8.39)
Median [Min, Max]	61.0 [47.0, 75.0]	63.0 [38.0, 76.0]	55.0 [49.0, 75.0]	57.5 [40.0, 78.0]	62.0 [38.0, 78.0]
**Smoking**					
No	6 (66.7%)	20 (57.1%)	5 (35.7%)	6 (30.0%)	37 (47.4%)
Yes	3 (33.3%)	15 (42.9%)	9 (64.3%)	14 (70.0%)	41 (52.6%)
**Alcohol**					
No	4 (44.4%)	28 (80.0%)	9 (64.3%)	8 (40.0%)	49 (62.8%)
Yes	5 (55.6%)	6 (17.1%)	5 (35.7%)	12 (60.0%)	28 (35.9%)
Missing	0 (0%)	1 (2.9%)	0 (0%)	0 (0%)	1 (1.3%)
**T_stage**					
T1	9 (100%)	0 (0%)	0 (0%)	0 (0%)	9 (11.5%)
T2	0 (0%)	35 (100%)	0 (0%)	0 (0%)	35 (44.9%)
T3	0 (0%)	0 (0%)	14 (100%)	0 (0%)	14 (17.9%)
T4	0 (0%)	0 (0%)	0 (0%)	20 (100%)	20 (25.6%)
**N_stage**					
N1	2 (22.2%)	2 (5.7%)	1 (7.1%)	2 (10.0%)	7 (9.0%)
N2	5 (55.6%)	21 (60.0%)	8 (57.1%)	12 (60.0%)	46 (59.0%)
N3	2 (22.2%)	10 (28.6%)	5 (35.7%)	5 (25.0%)	22 (28.2%)
N0	0 (0%)	2 (5.7%)	0 (0%)	1 (5.0%)	3 (3.8%)
**M_stage**					
M0	9 (100%)	34 (97.1%)	14 (100%)	20 (100%)	77 (98.7%)
M1	0 (0%)	1 (2.9%)	0 (0%)	0 (0%)	1 (1.3%)
**Stage**					
IB	0 (0%)	2 (5.7%)	0 (0%)	0 (0%)	2 (2.6%)
IIA	0 (0%)	1 (2.9%)	0 (0%)	0 (0%)	1 (1.3%)
IIB	2 (22.2%)	1 (2.9%)	0 (0%)	0 (0%)	3 (3.8%)
IIIA	4 (44.4%)	17 (48.6%)	1 (7.1%)	3 (15.0%)	25 (32.1%)
IIIB	3 (33.3%)	12 (34.3%)	11 (78.6%)	13 (65.0%)	39 (50.0%)
IIIC	0 (0%)	1 (2.9%)	2 (14.3%)	4 (20.0%)	7 (9.0%)
IV	0 (0%)	1 (2.9%)	0 (0%)	0 (0%)	1 (1.3%)
**Chemotherapy**					
No	0 (0%)	0 (0%)	0 (0%)	0 (0%)	0 (0%)
Yes	9 (100%)	35 (100%)	14 (100%)	20 (100%)	78 (100%)
**Radiotherapy**					
No	0 (0%)	3 (8.6%)	2 (14.3%)	1 (5.0%)	6 (7.7%)
Yes	9 (100%)	32 (91.4%)	12 (85.7%)	19 (95.0%)	72 (92.3%)
**Immunotherapy**					
No	8 (88.9%)	35 (100%)	13 (92.9%)	16 (80.0%)	72 (92.3%)
Yes	1 (11.1%)	0 (0%)	1 (7.1%)	4 (20.0%)	6 (7.7%)
**Death**					
No	8 (88.9%)	20 (57.1%)	6 (42.9%)	13 (65.0%)	47 (60.3%)
Yes	1 (11.1%)	15 (42.9%)	8 (57.1%)	7 (35.0%)	31 (39.7%)
**Progress**					
No	6 (66.7%)	10 (28.6%)	2 (14.3%)	8 (40.0%)	26 (33.3%)
Yes	3 (33.3%)	25 (71.4%)	12 (85.7%)	12 (60.0%)	52 (66.7%)

Supplementary Table S2. Sequences of primers for RT-qPCR assays

Gene	Primer sequence	Reaction system
ASCL1	Forward: GTCACAAGTCAGCGCCCAAG<br>Reverse: TGTAGCCAAAGCCGCTGAAG	SYBR® Premix Ex Taq™
IGKC	Forward: GCACCATCTGTCTTCATCTTCC<br>Reverse: GCGTTATCCACCTTCCACTGTA	
IGLC2	Forward: CTATCTGAGCCTGACGCCTG<br>Reverse: TGTAGGGGCCACTGTCTTCT	
IGHG3	Forward: CACCATCTTCATCACACTCTTCCT<br>Reverse: CCCTGCCCAATCATGTTCCTAT	
IGHA1	Forward: ACTACACGAATCCCAGCCAGG<br>Reverse: GGTAGGTGGAGTTGAGGGAGATG	
GAPDH	Forward: GGAGCGAGATCCCTCCAAAAT<br>Reverse: GGCTGTTGTCATACTTCTCATGG	

Supplementary Table S3. Antibodies used in immunoreaction-based assays

Name	Company	Catalogue number	Dilution
**Primary antibody**			
IgG1	Abcam	ab108969	IB 1:5000
IgG2	Abcam	ab134050	IB 1:5000
IgG3	Abcam	ab109761	IB 1:5000
IgG4	Abcam	ab109493	IB 1:1000
λ light chain	Abcam	ab124719	IB 1:20000
κ light chain	Abcam	ab124727	IB 1:10000
IgG heavy chain	Proteintech	16402-1-AP	IB 1:1000
β-actin	Affinity	AF7018	IB 1:10000
**Secondary antibody**			
Anti-Rabbit	Jackson	111-035-003	IB 1:10000

IB: immunoblot
